# Interaction between hedgehog signalling and PAX6 dosage mediates maintenance and regeneration of the corneal epithelium

**Published:** 2012-01-18

**Authors:** Romana Kucerova, Natalie Dorà, Richard L. Mort, Karen Wallace, Lucy J. Leiper, Christina Lowes, Carlos Neves, Petr Walczysko, Freyja Bruce, Paul A. Fowler, Ann M. Rajnicek, Colin D. McCaig, Min Zhao, John D. West, J. Martin Collinson

**Affiliations:** School of Medical Sciences, University of Aberdeen, Institute of Medical Sciences, Foresterhill, Aberdeen AB25 2ZD, UK

## Abstract

**Purpose:**

To investigate the roles of intracellular signaling elicited by Hedgehog (Hh) ligands in corneal maintenance and wound healing.

**Methods:**

The expression of Hedgehog pathway components in the cornea was assayed by immunohistochemistry, western blot and reverse-transcription polymerase chain reaction (RT-PCR), in wild-type mice and mice that were heterozygous null for the gene encoding the transcription factor, paired box gene 6 (*Pax6*).  Corneal epithelial wound healing and cell migration assays were performed after pharmacological upregulation and downregulation of the hedgehog pathway.  Reporter mice, mosaic for expression of the gene encoding β-galactosidase (*LacZ*), were crossed to *Pax6*^+/-^ mice, mice heterozygous for the gene encoding GLI-Kruppel family member GLI3, and *Pax6*^+/-^ *Gli3*^+/-^ double heterozygotes, to assay patterns of cell migration and corneal epithelial organization in vivo.

**Results:**

Corneal epithelial wound healing rates increased in response to application of Sonic hedgehog (Shh), but only in mice with wild-type Pax6 dosage.  Downregulation of Hedgehog signalling inhibited corneal epithelial cell proliferation.  *Pax6*^+/-^ corneal epithelia showed increased proliferation in response to exogenous Shh, but not increased migration. Desert hedgehog (Dhh) was shown to be the major endogenous ligand, with Shh detectable only by RT-PCR and only after epithelial wounding. The activity of phosphatidylinositol-3-OH kinase-γ (PI3Kγ) was not required for the increased migration response in response to Shh.  Nuclear expression of the activator form of the transcription factor Gli3 (which mediates Hh signalling) was reduced in *Pax6*^+/-^ corneal epithelia. *Pax6*^+/-^ *Gli3*^+/-^ double heterozygotes showed highly disrupted patterns of clonal arrangement of cells in the corneal epithelium.

**Conclusions:**

The data show key roles for endogenous Dhh signalling in maintenance and regeneration of the corneal epithelium, demonstrate an interaction between Pax6 and Hh signalling in the corneal epithelium, and show that failure of Hh signalling pathways is a feature of *Pax6*^+/-^ corneal disease that cannot be remedied pharmacologically by addition of the ligands.

## Introduction

Epithelial wound healing is a complex multifactorial process requiring the coordinated response of several tissue and cell types, controlled by multiple signaling pathways [1]. Failure of wound healing causes significant burdens to both individuals and society, and strategies for clinical pharmacological modulation of wound healing are a research priority. This is especially the case for the ocular surface, where a failure to repair acute or chronic wounds can lead not only to pain and infection, but also deterioration of sight.

The corneal epithelium is maintained in part by limbal stem cells (LSCs) at the corneal periphery, and there is a dynamic equilibrium of cell production, centripetal cell migration from the periphery to the center of the cornea, epithelial proliferation and stratification, and cell desquamation [2-5]. Mice that are heterozygous for the gene encoding the transcription factor paired box gene 6 (*Pax6*) show corneal abnormalities including fragility, thinning, and conjunctivalisation of the corneal epithelium, and inflammation as well as pannus in the corneal stroma, recapitulating symptoms of aniridia-related keratopathy (ARK) in human *PAX6^+/−^* patients [6,7]. Examination of patterns of β-galactosidase (*LacZ*) mosacisim in reporter mice showed normal centripetal migration of epithelial cells during adult corneal maintenance to be disrupted [8]. There are physiologic abnormalities of the cytoskeleton and cellular signaling pathways in the corneal epithelium that appear to mimic a chronic wound healing response [9,10]. However, the signaling factors controlling corneal maintenance, and their function in ARK, are still incompletely understood.

Mice that are heterozygous for the gene encoding GLI-Kruppel family member GLI3 (Gli3), one of three zinc finger transcription factors mediating gene expression in response to Hedgehog ligands, have relatively normal eyes, but mice that are double-heterozygous *Gli3^+/−^ Pax6^+/−^* show retinal and other ocular abnormalities that are significantly more severe than those seen with either gene mutation individually [11]. This suggests that modulation of the Hedgehog signaling pathways may change the disease phenotype in *Pax6^+/−^* individuals.

In mammals and birds there are 3 hedgehog ligands, Desert Hedgehog (Dhh), Indian Hedgehog (Ihh), and Sonic Hedgehog (Shh). These secreted signaling proteins act as morphogens in a dosage dependent manner and control numerous processes during embryonic development and adult homeostasis including the maintenance of stem cells [12]. Hedgehog ligands bind the cell surface receptor Patched-1 (Ptch1), releasing inhibition of Smoothened (Smo) and allowing transmission of hedgehog signaling, canonically converging on the activity of the Gli transcription factors. Suppressor of fused, Su(fu), acts as the major Hh dependent pathway suppressor by retaining Gli proteins in the cytoplasm [13]. Loss of Su(fu) function results in ligand-independent potent activation of the pathway [14].

A non-canonical Hh signaling pathway is mediated through Smo-dependent extracellular signal-related kinase 1 (ERK1) activation via Ca^2+^ signaling and subsequent elevated proliferation [15]. Phosphatidylinositol-3-OH kinase (PI3K) dependent protein kinase B (Akt) activation is essential for Shh signaling in chicken neural explants and mediates the activation of Gli proteins in 3T3 cells [16].

Saika et al. [17] previously reported a role for Shh in the cornea, in particular during wound healing. They described *Shh* expression in limbal epithelial stem cells of intact rat cornea and its transient expression in the migrating corneal epithelium 12 h post injury. Furthermore, application of exogenous recombinant mouse (rm) Shh significantly improved corneal epithelial wound healing rates. More recently it was shown that exogenous rmShh promotes neovascularisation of wounded rat corneas [18]. In this paper we have confirmed that the Hedgehog signaling modulates cell migration and proliferation in the corneal epithelium, but propose Dhh is the major endogenous corneal Hedgehog ligand, and that it acts through pathways that require normal Pax6 dosage during corneal epithelial maintenance and wound healing.

## Methods

### Mice

*Pax6^+/Sey-Neu^* (*Pax6^+/−^*) mice were maintained on the CBA/Ca genetic background by heterozygous mating and eyes were usually enucleated from *Pax6^+/+^* and *Pax6^+/−^* 8 to 10 week old littermates. H253 (*XLacZ*) mice were as described in Tan et al. [19]. The *Gli3* Extra toes mice (*Gli3^+/XtJ^*, henceforth *Gli3^+/−^*) were obtained from Prof. David Price (University of Edinburgh, Edinburgh, UK) and maintained on an inbred CBA/Ca background as for Zaki et al. [11]. *PI3Kγ^−/−^* mice [20] were as described in Zhao et al. [21]. Nude mice (CD1-*Foxn1^nu/nu^*) were purchased from Charles River (Ramsgate, UK). All experiments were performed in accordance with institutional guidelines and UK Home Office regulations and followed the ARVO Guidelines for treatment of laboratory animals.

### Chemicals and reagents

Shh peptide was from R&D system laboratories (Abingdon, UK). Cyclopamine was purchased from Calbiochem (Nottingham, UK). Anti-Gli-3, was from Santa-Cruz Biotechnology (Santa-Cruz, CA) and was validated by use of blocking peptide to quench the signal. The 5E1 anti-Shh monoclonal antibody was obtained from the Developmental Studies Hybridoma Bank maintained under the auspices of the University of Iowa (Iowa City, IA), and an anti-Shh goat polyclonal antibody from R&D systems. Anti-Dhh was from Abcam (Cambridge, UK). Anti-BrdU was a mouse monoclonal from Millipore (Watford, UK). Cell culture reagents were from Invitrogen (Paisley, UK).

### Boyden chamber migration assay

Eyes were incubated in a solution containing Dispase II (15 μg/ml Dispase II; Invitrogen, Paisley, UK; 17 μg/ml sorbitol) at 4 °C for 18 h. The corneal epithelium was peeled from the stroma and incubated in 0.05% trypsin for 5 min at 37 °C. The cells were then washed, dissociated and resuspended in serum-free SHEM (modified SHEM: DMEM F-12 + Glutamax; Invitrogen, Paisley UK), 0.5% DMSO, 0.5 μg/ml hydrocortizone, 0.1 mg/ml cholera toxin A (Sigma, Poole, UK), 50 μg/ml gentamicin (Invitrogen, Paisley, UK), and 1.25 μg/ml amphotericin B (Invitrogen, Paisley, UK).

Boyden chamber assays were performed according to the manufacturer’s protocol for 1 h at 37 °C with 96 well microchemotaxis chambers (Neuroprobe, Warwickshire, UK) and 5 µm pore polycarbonate membranes (Neuroprobe). Dissociated epithelial cells, (1×10^5^/well) were added to the upper chambers and serum-free medium containing Shh peptide, or medium alone was added to the lower chambers. Cells that did not migrate were subsequently removed by gentle scraping of the upper side of the membrane, whereas the remaining cells were fixed and stained using the Diff-Quik staining kit (Techlab, Blacksburg, VA), and the filters were mounted on a glass slide. Cell migration was assessed by counting migrated cells in ten fields of view per well at 40× magnification with light microscopy.

### Ex vivo whole eye wound healing

*Pax6^+/+^* and *Pax6^+/−^* mice were killed and eyes were wounded using a trephine blade 0.8 mm or 1.5 mm in diameter. The corneal epithelium was debrided from the wound with an opthalmological scalpel. Eyes were enucleated and placed into 1 ml of serum free corneal culture medium (CCM, 15 ml Keratinocyte Growth Medium; Cambrex, Cambridge, UK), 19 ml DMEM:F12, Mercaptoethanol, 1 mM Hepes, Gentamycin (Sigma), according to Hazlett et al. [22]. Where described the culture medium was supplemented with 5 μg/ml Shh peptide reconstituted in 0.1% BSA in PBS. The wound healing rate was measured by fluorescein sodium staining. Eyes were photographed and the wound size measured in Adobe Photoshop 7 (Adobe Systems, San Jose, CA). The diameter of the wound during a 24 h experiment was calculated. The initial wound diameter was measured (X_0_) and measurements at each time point (X_6_, X_12_, X_24_) were subtracted from this reference point. The values plotted represent average diameter of circular wounds within the experimental population. Diameters for individual wounds were calculated as an average of two approximately perpendicular measurements across a single wound.

### In vivo cyclopamine injection

Nude mice (*Foxn1^nu/nu^*) of approximate mass 25 g received a subcutaneous injection of 0.2 ml of 4 mg/ml cyclopamine (LC Laboratories, Woburn, MA) in 45% (2-hydroxypropyl)-beta-cyclodextrin (Sigma) vehicle. Control mice received vehicle only. After 1 h, all mice received 0.2 ml of 10 mg/ml bromodeoxyuridine (BrdU) intraperitoneally, and were killed 2 h later, followed by fixation of eyes for 2 h in paraformaldehyde and processing for histological analysis as described below.

### Histological analysis

Eyes were fixed in 4% paraformaldehyde (PFA) for 2 h, processed to wax and 7 μm sections were cut. Eyes were sectioned in an anterior-posterior plane to include the cornea and lens. Deparaffinised sections were washed in 100% ethanol, incubated with 3% hydrogen peroxide in methanol for 20 min to block endogenous peroxidise activity. Sections were then rehydrated in ethanol gradient and washed in PBS. Antigen retrieval was performed by boiling in 0.01 M citrate buffer, pH 6, for 20 min. Slides were washed with Tris-buffered saline (TBS: 100 mM Tris, 0.9% NaCl, pH 7.6) and blocked with 4% normal serum (species according to secondary antibody) in TBS, 0.3% BSA for 2 h at room temperature. Sections were incubated in primary antibodies overnight at 4 °C (Gli-3 as above, diluted 1:100) and BrdU (diluted 1:400). Slides were washed in TBS and then treated with secondary antibody (biotinylated rabbit anti-mouse IgG diluted 1:200 in blocking buffer. Slides were then treated with avidin-biotin complex (ABC; Dakocytomation UK Ltd., London, UK) for 30 min and washed with TBS. The bound antibody was then visualized by 3,3′-diaminobenzidine (DAB) stain (5.9 ml of 20 mM Tris, pH 7.6, 100 μl of 50 mg/ml DAB, 1 μl of H_2_O_2_). For negative control, primary antibody was omitted and for Gli3, the antibodies were incubated with their blocking peptides before addition to slides, to demonstrate specificity of signal. X-Gal staining was performed on whole mount tissue following Collinson et al. [23]. Mosaic patterns in corneas of 15-week old XLacZ X-inactivation mosaics were analysed as described previously [8,23] but ImageJ was used for the quantification (see Results section).

### In situ hybridization

Digoxygenin-labeled riboprobes were transcribed from linearized Shh BSll-SK^-^ and Ptch1 cDNA (a kind gift from Dr. Christopher Hayes, Medical Research Council Mouse Genome Centre, Harwell, UK) using the DIG RNA-labeling kit (T3/T7; Roche Diagnostics Ltd., Burgess Hill, UK). In situ hybridization was performed on 7-μm sections hybridized overnight at 65 °C in a chamber humidified with 50% formamide and 5×SSC (Sigma). Slides were washed twice to remove unbound probe with 5×SSC for 2 h at 65 °C then in 0.1× SSC for 15 min at room temperature then in 50 mM Tris-HCl, 150 mM NaCl, pH 7.5. Samples were blocked in 2% blocking reagent in 0.1 M Maleate buffer, pH 7.5, 150 mM NaCl for 30 min and incubated with alkaline phosphatase conjugated anti-DIG antibody, diluted 1:500 for 1 h at room temperature. Sections were washed for 10 min in 100 mM NaCl, 50 mM MgCl_2_, 1 mM levamisole, 100 mM Tris-HCl, pH 9.5 then immersed in a buffer containing 3.5 μl/ml 5-bromo-4-chloro-3indolyl-phosphate (BCIP, 9.4 mg/ml) and 5 μl/ml 4-nitroblue tetrazolium chloride (NBT, 18.75 mg/ml) at room temperature in the dark until color developed. Slides were then briefly washed in PBS, re-fixed in 4% PFA for 10 min, washed in PBS and mounted in 80% glycerol.

### RT-PCR

Corneas (4 per genotype and/or treatment) were pooled and homogenized. RNA was isolated using the PeqGOLD total RNA isolation kit (Peqlab, Southampton, UK), with reverse transcription using Superscript II (Invitrogen, Paisley, UK) according to the manufacturers’ instructions with oligo-dT and random primers. PCR was performed on cDNA using the primers described in Table 1, with annealing temperature of 58–62 °C (optimized for each primer set). All primers were design to span introns to avoid false positives due to residual genomic DNA.

**Table 1:** PCR primers used in this analysis.

**Gene**	**Forward Sequence 5′-3′**	**Reverse Sequence 5′-3′**	**Product Size (bp)**	**Annealing temp (°C)**
*Shh*	CCCTTTAGCCTACAAGCAGT	CCACTGGTTCATCACAGAG	232	57
*Dhh*	ACATCACCACGTCTGACC	GAACACCGGTGCAAAGTCAC	309	58
*Ihh*	ACGTGCATTGCTCTGTCAAGT	CTGGAAAGCTCTCAGCCGGTT	221	58
*Ptch1*	CTGCTGCTATCCATCAGCGT	AAGAAGGATAAGAGGACAGG	452	58
*Ptch2*	TGCCTCTCTGGAGGGCTTCC	CAGTTCCTCCTGCCAGTGCA	207	58
*Smo*	GTGATGATGAGCCCAAGAGA	AGGGGCAGAGTGGTGAAGC	422	58
*Gli1*	CAGGGAAGAGAGCAGACTGA	AGCTGATGCAGCTGATCCAG	251	60
*Gli2*	ACGACTTTCTCCACACCCTGC	TGATGTAAGCTACCAGCGAG	478	60
*Gli3*	ATCCGCTCATTGCACAGCAG	TGAAGCTCAATGCAGGGCTG	436	58
*Hprt*	CCTGTCGGATTACATTAAAGCACTG	GTCAAGGGCATATCCAACAACAAC	301	57
*Gsk3β*	CAG CCT TCA GCT TTT GGT AGC	TCCACCAACTGATCCACACCAC	750	58
*Pax6*	AGTTCTTCGCAACCTGGCTA	TGAAGCTGCTGCTGATAGGA	519	62.5
*Su(Fu)*	GCT CCA GGT TAC CGC TAT CG	GCT GCC CAA ACT GTC CTTGC	772	58

### Western blot

One dimensional denaturing 12% PAGE was used to detect proteins in cell lysate. Prior to gel electrophoresis lysed protein samples were heated at 95 °C for 5 min. Gels were blotted onto nitrocellulose membrane (GE Healthcare, Little Chlafont, UK) in transfer buffer using Bio-Rad Mini-Protean III system (Berkeley, CA) for 1.5 h at 100V at 4 °C. Membranes were then washed in TBS 0.3% Tween followed by pre-blocking in 10% milk in TBS 0.3% Tween for 1 h at room temperature before incubation in primary antibody diluted in 0.5% skimmed milk in TBS 0.05% Tween-20, overnight at 4 °C. Unbound primary antibody was washed off with TBS 0.05% Tween-20 for 3×15 min at room temperature followed by incubation in secondary (horseradish peroxidise-conjugated) antibody for 1 h. Excess of secondary antibody was washed in TBS, 0.05% Tween-20 for 3×15 min at room temperature. Detection was performed using Immobilon™ chemiluminescent HRP substrate (Millipore, Watford, UK) and Amersham ECL Hyperfilm (GE Healthcare, Little Chlafont, UK) according to the manufacturers’ instructions.

### Statistical analyses

Statistical analyses were using either Microsoft Excel (Mountain View, CA), or the 'R' statistics package, an open source software package based on the 'S' programming language, using the package 'stats' (R Development Core Team, 2008).

## Results

### The migratory response of corneal epithelial cells to exogenous Shh requires normal Pax6 dosage

**Figure 1:**
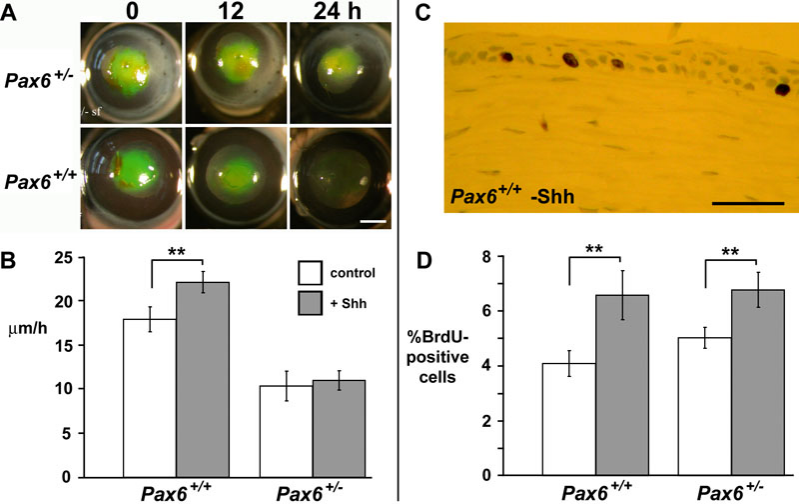
Exogenous Sonic hedgehog NH_2_-terminal peptide (rmShh-N) improves healing of wounded *Pax6^+/+^* but not *Pax6^+/−^* corneal epithelia. **A**: Representative views of wounded *Pax6^+/+^* and *Pax6^+/−^* eyes, following roughly circular corneal epithelial debridement, the wound visualized by topical application of sodium fluorescein after 0, 12, and 24 h of healing ex vivo. **B**: Mean±S.E.M. rate of epithelial wound closure for 8 wild-type and 5 *Pax6^+/−^* corneas with (gray bars) and without (white bars) application of 5 μg/ml rmShh-N. **C**: Representative image of a cluster of BrdU-positive nuclei (brown) in the basal layer of a wild-type control corneal epithelium. **D**: Mean±SEM percentage of BrdU-positive cells in the basal layer of the cornea epithelium of 3 *Pax6^+/+^* and *Pax6^+/−^* eyes, with (gray bars) or without (white bars) topical application of rmShh-N. *Pax6^+/−^* corneas increase their mitotic rate in response to Shh, but this does not translate to increased wound healing rate. ** represents p<0.01. Scale bars: **A**, 1 mm; **C**, 50 μm.

Topical application of Shh has already been reported to improve corneal epithelial wound healing in wild-types [17], but its effect on Pax6 mutants has not been investigated, in spite of its therapeutic potential for corneal disease. We investigated the stimulatory effect of Shh on corneal epithelial wound healing by determining the rate of wound closure in both *Pax6^+/+^* and *Pax6^+/−^* littermates in serum-free media supplemented with 5.0 μg/ml Shh. For each animal, one eye was treated in Shh and the contralateral eye was wounded concurrently and incubated in medium without Shh (Figure 1A). Exogenous Shh produced a small but significant increase in the rate of wound healing in *Pax6^+/+^* eyes 24 h post injury (control 17.9±1.4 μm/h, Shh 22.1±1.2 μm/h, n=8, paired *t*-test: p=0.007; Figure 1B). In contrast, *Pax6^+/−^* eyes showed a reduced rate of healing and no response to exogenous Shh (control 10.33±1.7 μm; Shh 10.99±1.1 μm/h; n=5; p=0.32; Figure 1B). Application of 200 μM cyclopamine in 1.5% DMSO, a treatment that in other systems has the effect of blocking the canonical hedgehog pathway by antagonising the Hedgehog protein co-receptor, Smoothened [24], did not significantly reduced the rate of wound closure after 24 h in wild-types (17.7±2.8 μm/h; n=6). Therefore our data suggest that although exogenous Shh accelerates wound healing in wild-type corneas, endogenous hedgehog signaling is not required for normal wound closure.

### Exogenous Shh increases corneal epithelial proliferation irrespective of Pax6 dosage

**Figure 2:**
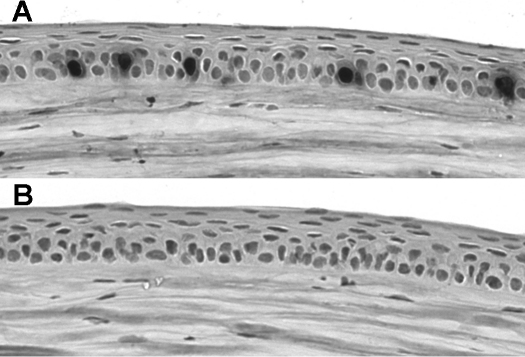
Cyclopamine completely inhibits corneal epithelial proliferation in vivo. **A**: Immunostaining of corneal epithelium 2 h after injection with BrdU in control mouse injected with vehicle only. Five BrdU-postive nuclei are labeled. **B**: Immunostaining of corneal epithelium 2 h after injection with BrdU in mouse injected with 32 mg/kg cyclopamine. No BrdU-positive nuclei are detectable, and no positive nuclei were detected in any section of the corneas of 3 cyclopamine-treated mice.

Hedgehog signaling can act as a mitogen, which in part underlies the oncogenic activity of the signaling pathway. The potential role of exogenous Shh for increasing corneal epithelial proliferation after wounding was investigated. Corneal epithelia were wounded as described above and allowed to heal with or without exogenous Shh as above. Bromodeoxyuridine (BrdU; 10 mg/ml) was added to the medium for 6 h before fixing the tissue to assay proliferation (Figure 1C,D). Exogenous Shh increased the rate of cell proliferation in the basal layer of the wild-type corneal epithelium: 4.07±0.47% of cells were BrdU-positive in controls in serum-free medium, compared to 6.56±0.89% in Shh-treated corneas (n=3 of each genotype; Mann–Whitney U test, p=0.009). Shh also increased proliferation rate in *Pax6^+/−^* corneas after wounding, from 5.01±0.38% to 6.75±0.64% (n=3 of each genotype; Mann–Whitney U test, p=0.006) showing that *Pax6^+/−^* corneal epithelia can respond to Shh application by increasing their proliferation rate. In vivo, injection of adult mice with 32 mg/kg cyclopamine completely inhibited corneal epithelial cell proliferation (Figure 2; n=3 cyclopamine-treated, 3 controls). Hence pharmacological upregulation or downregulation of Hedgehog signaling, which leads to increased or decreased mitosis, does not necessarily translate to a increase or decrease in wound healing rate (as shown above). It was concluded that the effect of Shh application on increasing the early stages of healing in wild-type corneas is not likely to be a function of its mitogenic effects. Although *Pax6^+/−^* corneal epithelia increase their proliferation rate when Shh is applied, this has no effect on their rate of wound healing.

### Shh promotes corneal epithelial cell migration

**Figure 3:**
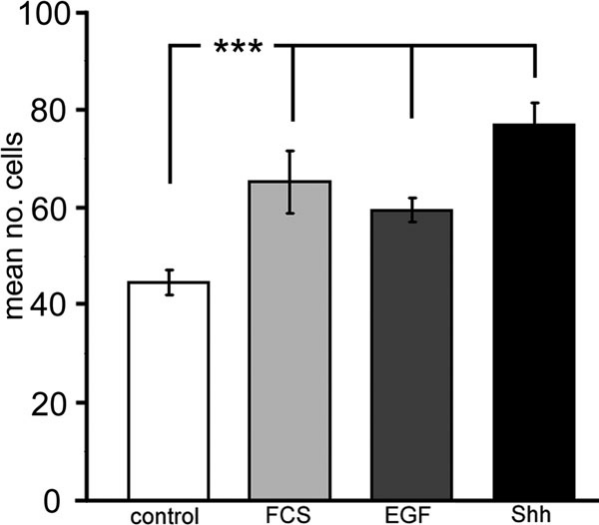
Effect of exogenous Shh on in vitro corneal epithelial cell migration. Mean number (±S.E.M) of wild-type corneal epithelial cells per ten fields of view migrating in 60 min across Boyden chamber membrane toward a source of serum-free corneal culture medium (sfCCM; ‘control’), CCM with 10% fetal calf serum (FCS), sfCCM with 50 ng/ml mouse epidermal growth factor (EGF) or sfCCM with 5 μg/ml rhShh-N (Shh). *** represents p<0.001.

Data above suggested that changes in wound healing rates seen in wild-type corneal epithelia following Shh application were unlikely to be due to an effect on cell proliferation. We hypothesized that Shh directly increases the migratory potential of corneal epithelial cells. This was investigated for dissociated corneal keratinocytes in a Boyden chamber migration assay. It was found that wild-type mouse corneal epithelial cells migrated toward Shh (5 μg/ml), which produced a 1.72 fold increase in migration compared with control serum-free media (n=27 replicates, p<0.001; Figure 3). Epithelial cells also migrated toward other growth factors including EGF (50 ng/ml), which produced a 1.33 fold increase over serum-free control (Figure 3, n=12, p=0.0002). These data were comparable to the effect of serum or FGF (50 ng/ml) that is known to stimulate epithelial cell migration.

This experiment showed that Shh stimulates the migration of wild-type corneal epithelial cells. *Pax6^+/−^* corneal epithelial cells could not be tested in the Boyden chamber for technical reasons (see Discussion).

### Activity of PI3Kγ is not required for the increased healing rate after Shh addition

It was shown previously that Shh peptide can induce PI3K-dependent activation of Akt [16,25,26]. PI3Kγ has previously been shown to be necessary for optimal corneal epithelial cell migration [21]. We examined wound healing in *PI3Kγ^−/−^* mice, with and without application of exogenous 5 μg/ml Shh. The results are not directly comparable with our other data because the mice are on a different genetic background (C57BL/6) but wound healing in control serum-free medium was slow (13.4±3.6 μm/h). In contrast, with application of Shh the rate of epithelial wound healing over 12 h rose significantly to 28.8±4.7 μm/h (*t*-test: p=0.014). Hence although application of Shh activates Akt, PI3Kγ is not required for the accelerated wound healing response to exogenous Shh.

### Expression of Shh signaling pathway components in corneal epithelium

**Figure 4:**
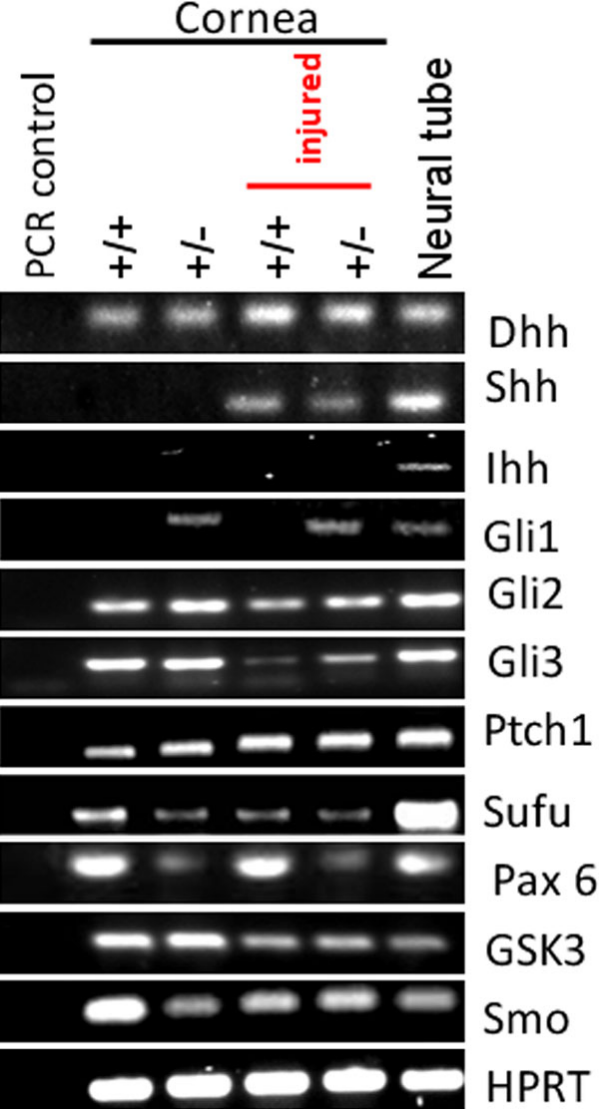
RT–PCR analysis of Hedgehog signaling pathway genes in wounded and unwounded wild-type and *Pax6^+/−^* corneas. RT–PCRs (primers and conditions as described in the Methods section and Table 1) were performed on pooled samples of wounded and unwounded wild-type and *Pax6^+/−^* corneas (4 corneas from different mice pooled for each condition). Wild-type and *Pax6^+/−^* corneas are represented as ‘+/+’ and ‘+/−’ respectively. Negative controls were PCRs on corneal samples without reverse transcriptase but otherwise treated as the other samples. Positive control was RT–PCR from preparations of embryonic neural tube and surrounding dorsal structures including the notochord. Multiple signaling pathway components were detected. The only difference between wounded and unwounded corneas was the upregulation of *Shh*. The only difference between wild-type and *Pax6^+/−^* was the upregulation of *Gli1*.

Multiple canonical Shh signaling components were detected by RT–PCR in wild-type and *Pax6^+/−^* corneas, before and after injury (Figure 4), suggesting that the failure of *Pax6^+/−^* corneas to increase wound healing rate in response to Shh application was not attributable to failure of pathway gene expression. By RT–PCR we were able to demonstrate mRNA for Dhh in uninjured wild-type and *Pax6^+/−^* adult corneas, but no expression of the genes encoding Shh or Ihh ligands (Figure 4). These data are consistent with an earlier study of Hedgehog family ligands in adult ocular tissues [27] and suggest that Dhh is the predominant ligand. However, *Shh* expression was induced in wild-type and *Pax6^+/−^* corneas assayed 2 h after injury. Expression of receptors Ptch1 and Smo and the signaling intermediates Su(fu) and GSK3β were detected in all adult corneal preparations (Figure 4). Canonical Shh signaling is mediated by Gli family transcription factors, and we were able to demonstrate expression of Gli2 and Gli3, but not Gli1 in the wild-type cornea by RT–PCR. In contrast to wild-type, *Gli1* expression was detected in uninjured and injured *Pax6^+/−^* corneas. Gli1 upregulation is normally taken as an indicative readout of active Hh signaling, and the significance of this observation is not yet known.

**Figure 5:**
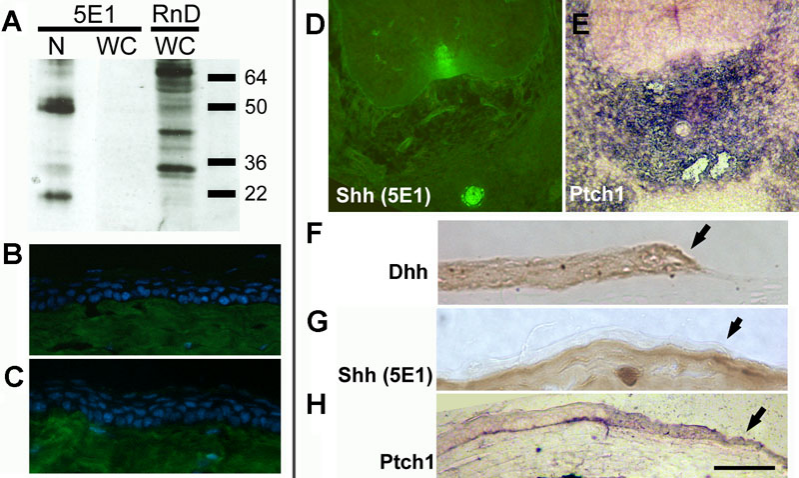
Expression of Hedgehog signaling pathway components in the adult corneal epithelium. **A**: western blot analysis of embryonic notochord/neural tissue (N) or wounded adult corneas using the 5E1 mouse anti-Shh-N monoclonal (Developmental Studies Hybridoma Bank) or R&D Systems goat anti-mShh-N polycolonal AF464. 5E1 recognizes 2 major bands at sizes predicted for Shh isoforms in embryonic tissue (left hand lane), but detects no Shh-N in the wounded adult cornea (middle lane). The goat polyclonal recognizes multiple non-Shh bands in wounded corneas. **B**, **C**: No Shh is detected by immunohistochemistry in the wild-type adult corneal epithelium by the 5E1 antibody (**B**), although there is strong background staining of the underlying corneal stroma by the Alexa-488 conjugated goat anti-mouse IgG_1_ secondary antibody (A21121; Molecular Probes), as shown in **C** the negative (no primary antibody) control. **D**: Shh localization in transverse section of E12.5 notochord and floor plate (immunohistochemistry using 5E1 monoclonal antibody) and **E** *Ptch1* expression (purple) upregulated in tissues around the notochord shown by in situ hybridization. The sense control was blank (data not shown). **F**: Localization of Dhh by immunohistochemistry with DAB (brown) endpoint in the wounded corneal epithelium. Wound edge is indicated by an arrow. **G**: No Shh in the corneal epithelium at a healing wound (arrow) edge shown by immunohistochemistry using the 5E1 monoclonal antibody. **H**: Upregulation of *Ptch1* at the wound edge (arrow) is evidence that Hedgehog signaling is active. Scale bar: 100 μm.

Although Shh message was detectable by RT–PCR in wounded corneas, expression must be at very low levels because western blot of corneal epithelia, wounded in vivo, detected no Shh protein using the 5E1 anti-Shh monoclonal antibody (Developmental Studies Hybridoma Bank; Figure 5A). Major bands at around 50 kDa and 22 kDa corresponding to the uncleaved and NH_2_-terminal Shh peptides were detected from embryonic notochord and neural tube. The antibody used in previous work (goat anti-mShh; AF464; R&D Systems) [17], was found to be non-specific. Western blotting of wounded corneas with this antibody yielded 16 bands between 64 and 16 kDa, and neither of the major bands corresponded to known sizes of Shh peptides. No Shh protein was detected in the corneal or limbal epithelia using the 5E1 monoclonal antibody (Figure 5B,C) in contrast to strong signal in the embryonic notochord and floor plate (Figure 5D). In situ hybridization showed Ptch1 upregulation in mesenchyme surrounding the notochord (an assay for cells where Hh signaling is active; Figure 5E). At the healing edge of wounded corneal epithelia, Dhh protein was detected but no Shh labeling was visible using the 5E1 monoclonal antibody (Figure 5F,G). There was some evidence for weak Ptch1 upregulation, suggesting active Hh signaling (Figure 5H). It was concluded that there is little endogenous limbal or wound edge localization of Shh protein in adults and that Dhh is probably the major corneal Hedgehog family ligand.

**Figure 6:**
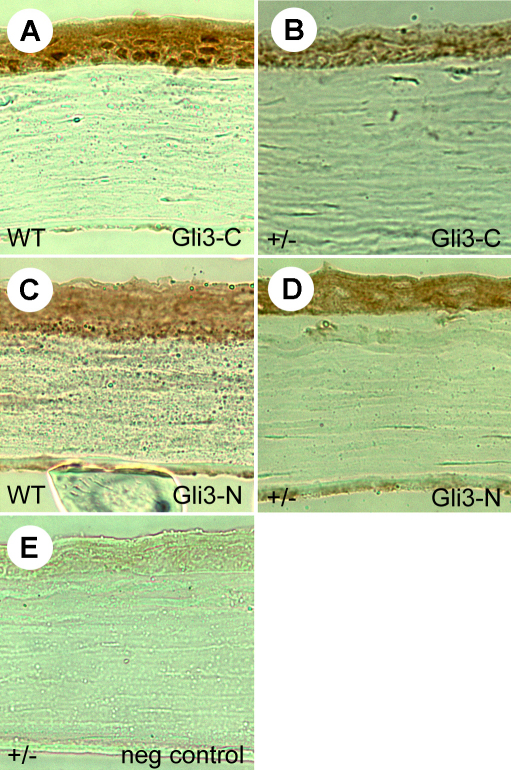
Gli3 localization in wild-type and *Pax6^+/−^* corneal epithelia. **A-D**: Localization of Gli3 protein in adult *Pax6^+/+^* (WT; **A**, **C**) and *Pax6^+/−^* (+/−; **B**, **D**) corneal epithelia using antibodies directed against the activator COOH-terminal fragment and the repressor NH_2_-terminal form of the protein. Nuclear COOH-terminal protein is present in wild-type epithelial nuclei but is not easily detectable in *Pax6^+/−^* corneas. **E**: The negative control is with no primary antibody. Scale bar: 50 μm.

Immunohistochemistry using an antibody directed at the COOH-terminus of Gli3 confirmed that activator form of Gli3 was expressed predominantly in cell nuclei of the corneal epithelium (Figure 6A). Nuclear staining appeared weaker in *Pax6^+/−^* littermates (Figure 6B). The NH_2_-terminal product of Gli3 was localized in cytoplasm of *Pax6^+/+^* epithelial cells but was also weaker in *Pax6^+/−^* littermates (Figure 6C,D).

### Gli3 deficiency exacerbates the corneal migration defect resulting from Pax6 heterozygosity

Immuohistochemistry suggested that Gli3 nuclear localization was disrupted or absent in *Pax6^+/−^* corneal epithelia. Previously, we and others have shown that although *Gli3^+/−^* mice have only very subtle eye defects, *Pax6^+/−^ Gli3^+/−^* double heterozygotes have ocular defects that are much more severe than either single heterozygote phenotype [11]. To determine whether heterozygousity for *Gli3* affects corneal epithelial cell migration, both mutations were bred onto the H253 *XLacZ* background. This reporter mouse carries an X-linked *LacZ* transgene under the control of a housekeeping promoter. Due to X-inactivation during early embryogenesis, females show mosaic β-galactosidase expression in all tissues [19]. Their corneas display a radial pattern of blue striping that appears to reflect centripetal migration of streams of cells from the limbus during adult life, and has previously been shown to be disrupted in Pax6 heterozygotes [4,8,23].

**Figure 7:**
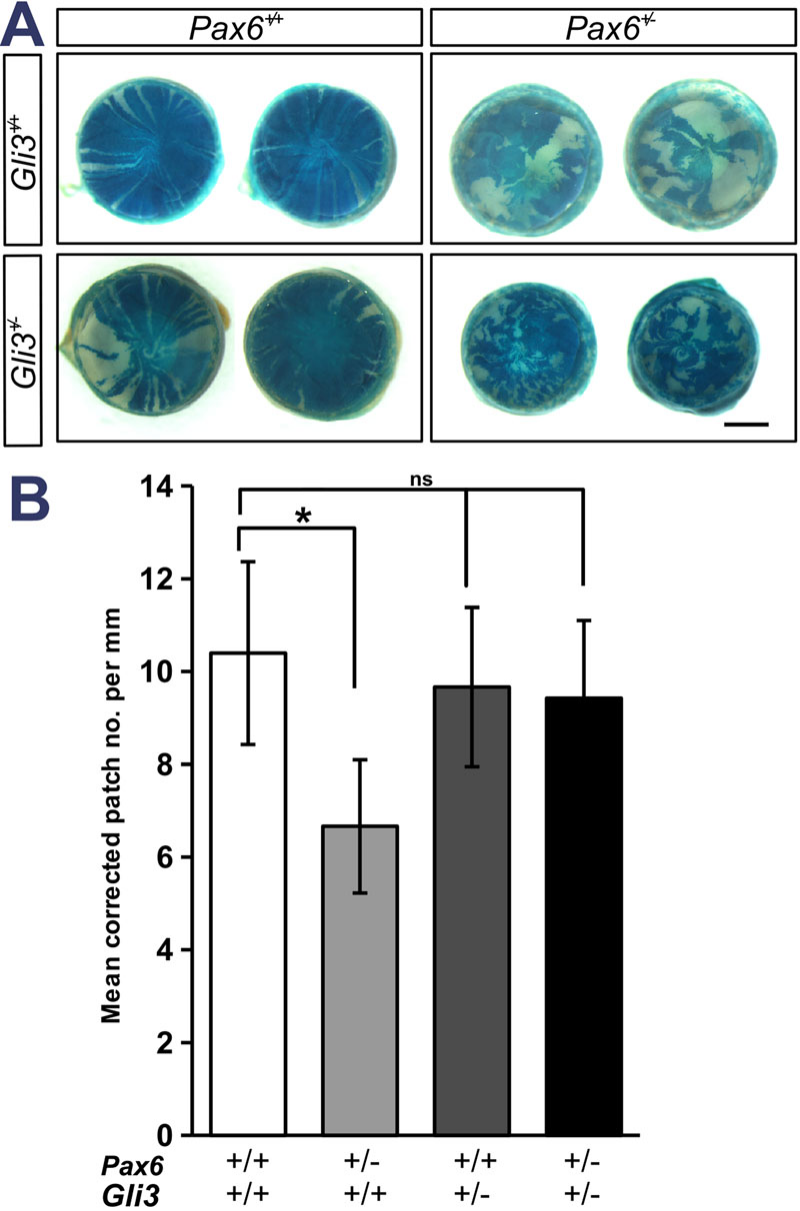
Disrupted clonal patterns of corneal epithelial cells in *Pax6*^+/−^/*Gli3*^+/−^ double heterozygotes. **A**: Representative images of X-Gal stained corneal epithelia from *XLacZ* transgenic females that are either wildtype, heterozygous for *Pax6* or *Gli3* alone, or double heterozygotes. The normal patterns of radial striping in wild-types and disrupted striping in *Pax6****^+/−^*** have been published previously [8,23]. *Pax6****^+/+^*** *Gli3****^+/−^*** corneas were found to be relatively normal, but *Pax6****^+/−^*** *Gli3****^+/−^*** double heterozygotes were very highly affected, with loss of striping and presence of much smaller disrupted patches of cells. **B**: The corrected mean patch number around the circumference, an estimate of the number of active clones of limbal stem cells, in corneas of different genotypes expressed per millimeter of the corneal circumference as mean±C.I. Scale bar in **A**=1 mm; * represents p<0.05; ns=not significant.

Hemizygous *XLacZ^Y/+^ Gli3^+/−^* male mice were mated to *Pax6^+/−^* females and the resulting female littermates were genotyped and their eyes stained with XGal at 15 weeks of age. At least 10 mice of each genotype were analyzed. The pattern of corneal epithelial staining in *Pax6^+/+^ Gli3^+/−^ XLacZ* adults was found to be grossly similar to *Pax6^+/+^ Gli3^+/+^ XLacZ* littermates (Figure 7A). That of *Pax6^+/−^ Gli3^+/+^ XLacZ* adults showed irregularities in the radial striping, of variable severity, as described previously. In contrast, patterns of *LacZ* expression in *Pax6^+/−^ Gli3^+/−^ XLacZ* double heterozygotes were very highly disrupted with a patchwork organization and little evidence in some cases of any radial orientation or migration at all (Figure 7A). Wild-type *XLacZ* corneal epithelia often display a spiral pattern of cell migration as noted previously [23], but in one *Pax6^+/−^ Gli3^+/−^* case there was a double spiral at the center (right hand *Pax6^+/−^ Gli3^+/−^* eye in Figure 7A) which suggested particular instability of cell migration patterns. By counting the mean number of blue and white stripes and correcting for the total proportion of blue corneal area it is possible to estimate the number of coherent clones of limbal epithelial cells (LSCs) that maintain the corneal surface [8,28]. Analysis of the corrected patch number around the circumference of the four genotypes generated from the above cross was performed using the freeware image analysis software package ImageJ with the 'Clonal Tools' plugin described in [28]. The analysis showed a statistically significant trend when the corrected patch number (expressed per millimeter of corneal circumference) was compared by one-way ANOVA (p<0.02). Subsequent post-hoc tests revealed no significant change in the number of corrected patches per millimeter of the corneal circumference in *Pax6^+/+^ Gli3^+/−^* adults (Mean±95% confidence interval [C.I.]: 9.66±1.72; n=16) compared to wild-type litter-mates (10.4±1.97; n=17; Tukey HSD: p=0.96). However the corrected patch number in the *Pax6^+/−^ Gli3^+/+^* single heterozygotes (6.66±1.44; n=27) was lower than for wild-type or *Pax6^+/+^ Gli3^+/−^* single heterozygous littermates although the difference only reached statistical significance for the comparison with wild-types (Tukey HSD: p=0.024; Figure 4B). The lack of clear stripes in *Pax6^+/−^ Gli3^+/−^ XLacZ* double heterozygotes made the analysis less easy to interpret but counting the number of blue and white patches of cells at the corneal circumference produced an estimate of 9.43±1.67 (n=31) corrected patches in the double heterozygotes; this was not statistically significant when compared to the wild-type group, (p=0.87).

The data confirm a functional genetic interaction between Hedgehog signaling and Pax6 dosage in the corneal epithelium. Because the *Pax6^+/−^ Gli3^+/−^ XLacZ* double heterozygotes do not always form stripes a change in the number of active LSCs can not be inferred however it is clear from the data that *Gli3^+/−^* heterozygotes have migratory defects that are only visible on the *Pax6^+/−^* background and are distinct from animals that are heterozygous for *Pax6^+/−^* or *Gli3^+/−^* alone. This is consistent with evidence for a genetic interaction between Pax6 and hedgehog signaling in the retina [11].

## Discussion

Multiple molecular pathways can modulate the reorientation, migration, and stratification of cells during epithelial wound healing. In this study we confirmed that application of exogenous Shh can significantly improve corneal wound healing rates, but that normal Pax6 dosage was required, and that there is a genetic interaction between Hh signaling and Pax6 function in the cornea. However, we found that Hedgehog signaling is not required for wound healing, and that furthermore Shh is only expressed in the wounded cornea at very low levels (detectable by RT–PCR but not immunohistochemistry or western blot). Takabatake et al. [27] detected Dhh but not Shh in mouse corneas by RT–PCR, a result consistent with our data. Hedgehog-Gli signaling can promote mitogenic effects via transcriptional and spatial control of N-Myc [29,30], and we predicted that this would represent the mechanism for a potentially therapeutic effect of exogenous Shh, however this appears not to be the case: whereas both wild-type and *Pax6^+/−^* corneas showed an increase in mitosis when exposed to exogenous Shh, only wild-type corneas also showed an increased wound healing rate. Our demonstration that cyclopamine application completely inhibits corneal epithelial proliferation but has no influence on the immediate wound healing rate. Furthermore, application of Shh was shown to directly increase the migratory potential of corneal keratinocytes in the in vitro Boyden chamber assay – *Pax6^+/−^* cells did not migrate at all in this assay, but we are unable to determine whether this is a direct biologic effect of reduced Pax6 dosage or a downstream consequence of the susceptibility of these cells to trituration. *Pax6^+/−^* cells can migrate well as monolayers in vitro and can respond positively to application of epidermal growth factor [31]. We conclude (consistent with our Boyden chamber assay) that Hedgehog signaling is one of several semi-redundant pathways that can stimulate epithelial cell migration but which has only a minor role in the normal healing response. Our data are consistent with a model whereby endogenous Hh signaling maintains the corneal epithelium through increasing the mitotic index and the migratory potential of epithelial cells, acting via Smoothened and then downstream via PI3K-Akt-mediated and Gli-mediated signaling pathways. Application of exogenous Shh pharmacologically aids wild-type wound healing, but has no therapeutic effect on *Pax6^+/−^* cells. Thus although pharmacological modulation of Hh may aid wound healing in some corneal diseases resulting from injury, infection or genetic defect, it would appear not to be a viable therapy for human aniridia.

Observations of cell migration and radial striping of epithelia in uninjured corneas do demonstrate that migration from the limbus to the corneal center does occur [4]. The patchwork pattern of β-galactosidase activity in our *Pax6^+/−^ Gli3^+/−^ XLacZ* adult mice is therefore intriguing. In some respects this pattern is similar to that observed in wild-type mice at 0–3 weeks of age [23], which is presumed to reflect interstitial growth throughout the corneal epithelium during development, before LSC activation. Why this patchwork expression is maintained in adults that are double heterozygotes is unknown. It may be that clonal populatons of cells are more likely to break up (i.e., lose their coherence) in the double mutants, giving disrupted patches of cells. It was suggested previously that there is a minor LSC deficiency in *Pax6^+/−^* adults [8]. It is possible that adding *Gli3* heterozygosity to reduced Pax6 dosage exacerbates the LSC deficiency and forces the corneal epithelium into self-maintenance by more random growth independent of LSC input, and that this explains the lack of radial stripes in these corneas. A similar explanation has been proposed for the maintenance of the corneal epithelium in *Dstn^corn1/corn1^* homozygotes [32] and a similar phenotype has been reported for corneas of *XLacZ* mosaic mice that are heterozygous for the *Pax6^Leca4^* missense mutation [33]. Whether Hh signaling in the cornea has any role for the activity of stem cells will be the subject of future study.
